# Uncommon Presentation of a Perforated Appendicitis Leading to Duodenal Fistula: Case Report and Literature Review

**DOI:** 10.1155/2024/8269752

**Published:** 2024-05-29

**Authors:** Jad El Bitar, Hani Maalouf, Souad Ghattas, Ribal Aby Hadeer, Ahmad Younes, Hind Rahban, Ziad El Rassi

**Affiliations:** ^1^ Department of General Surgery Balamand University Saint Georges Hospital University Medical Center, Beirut, Lebanon; ^2^ Laboratory Department Lebanese American University Medical Center, Beirut, Lebanon; ^3^ General Surgery Department Saint Georges Hospital University Medical Center, Beirut, Lebanon

## Abstract

Multiple types of fistulas associated with the appendix have been reported; however, duodenal fistula resulting from perforated acute appendicitis has only been documented in one previous case. In this report, we present the case of an 18-year-old male patient who was diagnosed to have a complicated appendicitis in its normal position with abscess formation. He was started on IV antibiotics and underwent a CT-guided drainage of the abscess with drain placement. Two days later due to biliary output from the drain, CT fistulography and diagnostic laparoscopy were performed that revealed the presence of a duodenal fistula. The potential for duodenal fistula formation in patients with complicated appendicitis must always be taken into consideration. Consequently, it is crucial to establish an appropriate management plan aimed at preventing additional serious complications arising from duodenal perforation.

## 1. Introduction

Acute appendicitis stands as one of the most prevalent surgical emergencies globally, carrying a lifetime risk of 8.6% in men and 6.7% in women [1]. Although it generally manifests with symptoms like nausea, fever, and pain in the right lower quadrant, its presentation can be atypical, potentially leading to delayed diagnosis [1]. Such delays may in turn give rise to complications like appendiceal perforation, abscess formation, intra-abdominal adhesions, sepsis, and, in severe cases, even mortality [1].

Multiple types of fistulas associated with the appendix have been reported, but duodenal fistula due to perforated acute appendicitis is only reported in one previous case [2].

We hereby present an unusual case involving a patient with perforated appendicitis and an associated abscess collection. Notably, a duodenal fistula was identified during the process. In this report, we detail the optimal management approach employed to address this complex scenario.

## 2. Case

### 2.1. Clinical Presentation

An 18-year-old male patient, with no previous medical and surgical history, presented to our emergency department with five-day duration of diffuse abdominal pain. It was associated with mild nausea and multiple episodes of nonresolving low-grade fever. He reported no vomiting, diarrhea, anorexia, or weight loss and no previous episodes of abdominal pain. The patient consulted his general practitioner before presenting to us who started him on ciprofloxacin and metronidazole with no improvement after 3 days.

### 2.2. Physical Examination

On admission to the emergency department, the patient was still febrile. His physical exam revealed a soft abdomen on palpation, slightly distended, with diffuse abdominal pain and a maximal point tenderness localized to the right lower quadrant.

### 2.3. Investigations and Treatment Plan

Laboratory studies showed leukocytosis, namely, a white blood cell count of 19,000/mm^3^, otherwise unremarkable results.

**Figure 1:**
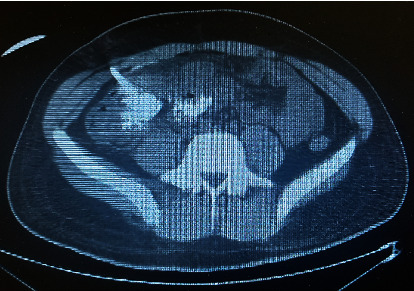
CT-guided drainage of the 10 cm abscess collection near the appendix.

A computed tomography (CT) abdomen pelvis scan with intravenous (IV) contrast showed the presence of an inflamed appendix with a 10 cm abscess collection. The patient was diagnosed with complicated appendicitis associated with an abscess collection. He was started on IV antibiotics and underwent a CT-guided drainage of the abscess with drain placement (Figure 1). CT fistulography postdrain insertion showed good drainage of the collection with no leak.

**Figure 2:**
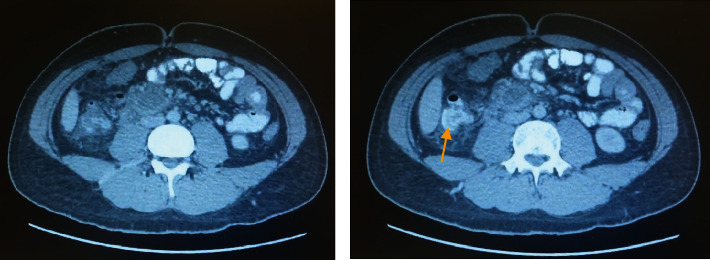
CT fistulography showing fistulous tract between the collection and the third portion of the duodenum (yellow arrow).

After starting this conservative treatment, his condition generally improved. However, two days later, the drainage output changed in color and appeared to be biliary in nature. A repeat CT scan abdomen pelvis showed a wider collection. Upon administration of contrast material into the drain, there was a passage of contrast through the third portion of the duodenum into the proximal small bowel, signaling a fistulous tract between the collection and the third portion of the duodenum. It was also noted that there was thickening of the wall of the proximal jejunum and the third and fourth portions of the duodenum raising the possibility of inflammatory bowel disease (Figure 2). This could also explain the fistula formation due to another cause. However, colonoscopy ruled out the presence of inflammatory bowel disease.

A diagnostic laparoscopy was performed that revealed the presence of a contained right lower quadrant duodenal fistula surrounded by the omentum, and the area was seen to be drained by the radiological drain. No distant collection or peritonitis was observed, so a decision was made to go for conservative management. It included keeping the patient with no oral intake, placing the patient on total parenteral nutrition, and IV antibiotics until stabilization of his general condition that was 3 weeks after admission. The patient was followed closely clinically and medically. The drain output was between 10 and 20 cc daily, and it was a low-output fistula. He was discharged home on the third week postadmission on two additional weeks of PO antibiotics, and the drain was removed later on as outpatient when the output zeroed two weeks after discharge.

### 2.4. Follow-Up

Eight weeks following drainage, the patient underwent laparoscopic appendectomy. The surgery was uneventful and uncomplicated. Pathological examination showed a perforated appendix with features of acute appendicitis and no evidence of tumors or other findings.

## 3. Discussion

Acute appendicitis is a common disease among all age groups, particularly in young adults [3]. It is thought to be caused by luminal obstruction from various etiologies [4]. Lymphoid hyperplasia is the most common underlying condition in the first 20 years and fecal obstruction in elderly patients [4]. Other rare causes have been also described ranging from malignant to benign neoplasm such as tubule-villous adenoma [5]. A delay in diagnosis may lead to fatal conditions with an increase in morbidity and mortality. Therefore, making an early diagnosis is crucial [3]. A perforated appendicitis may present with many different conditions, including abdominal mass, abscess, peritonitis, and rarely fistulas. An intra-abdominal abscess is the most common complication following a perforated appendicitis, with an incidence rate of 14 to 18% [6]. Duodenal fistula due to acute appendicitis is exceptional with only one case reported so far in literature [2]. Fistulas with colon, umbilicus, skin, kidneys, bladder, and aorta have previously been described [3].

Acute appendicitis complicated by duodenal perforation should be differentiated from Valentino's syndrome mimicking acute appendicitis. This condition is described as fluid leaking from a perforated gastric or duodenal ulcer that may induce peritonitis if it drains down the right paracolic gutters and spread to the appendix. This will cause chemical irritation that mimics acute appendicitis [7]. In the presented case, abscess formation and duodenal fistula were considered as an acute complication of perforated appendicitis. CT scan showed a perforated appendicitis with 10 cm abscess collection, and no pneumoperitoneum was seen that can suggest gastric or duodenal perforation.

Delayed diagnosis is usually the case with atypically located cases of fistulas. Even with the support of advanced radiological procedures, the fistula is not easy to be found [3]. CT fistulography is appreciated for the localization of the fistula, but the gold standard is an exploratory laparoscopy. It helps in confirming this unusual disorder, and concurrently, it allows for the treatment in some cases by appendectomy and excision of the fistulous tract [3]. According to the initial presentation of our patient, he was treated with drainage of the abscess collection. Two days later, due to bilious drainage, the diagnosis was confirmed with CT fistulography and diagnostic laparoscopy.

The management of duodenal fistulas is puzzling due to the high enzyme-rich output and anatomical location. There is a daily influx of around 10 liters of gastric, biliary, and pancreatic fluids that pass through the duodenum [2]. Successful management usually depends on control of sepsis, good nutritional support, and maintaining intestinal continuity. Ultimate closure of the fistula will be ensured. Invasive and noninvasive techniques can be adapted for the management.

Invasive interventions can be illustrated first by diversion techniques. Stents can be installed percutaneously or endoscopically for high output duodenal fistula diversion [8]. Another technique is the repair with tissue adhesive agents deployed by interventional radiology [8]. Finally, surgical interventions were described, namely, decompression with Puestow-Olander tube and diversion with duodenostomy. Simple closure with patch is another alternative. The patches can be omental, rectus abdominal, or serosal. Lastly, resection and anastomosis are also options along with bypass with mainly Roux-en-Y that can be used in large duodenal defects [8]. However, when there is sepsis, surgical interventions are more likely to be unsuccessful. Surgeries are left only as an option when the output of the fistula is very high or when conservative interventions have failed [8].

Babu and Finch concluded that the conservative management by administering total parenteral nutrition and withholding oral nutrition for 4-6 weeks was associated with a success rate between 25 and 75%. Further interventional approach is appropriate after this period or once the patient has nutritionally recovered to support a major intervention [8]. Dealing with our case, we opted for conservative management since the patient was hemodynamically stable and he was responding to the medical treatment of antibiotics, optimal parenteral nutrition, and drainage. In addition, the fistula was well controlled with a proper drainage, having a low output, and it was contained and did not spread in the abdomen. Consequently, this young patient was spared from a more invasive procedure. An interval appendectomy after fistula closure was a very straightforward surgery to perform, and it seemed inevitable due to the perforation and to prevent further future complications.

## 4. Conclusion

In summary, we present here an extremely unusual complication of perforated appendicitis. The possibility of duodenal fistula formation in patients with complicated appendicitis must be considered. Early diagnosis, subsequently, will help in providing the ideal treatment to prevent further hazardous complications.

## Data Availability

All data are available upon request at the Department of General Surgery, Saint George Hospital University Medical Center, University of Balamand, Beirut, Lebanon, where the work was done.
